# Atypical aetiology in patients hospitalised with community-acquired pneumonia is associated with age, gender and season; a data-analysis on four Dutch cohorts

**DOI:** 10.1186/s12879-016-1641-9

**Published:** 2016-06-17

**Authors:** Vivian M. Raeven, Simone M. C. Spoorenberg, Wim G. Boersma, Ewoudt M. W. van de Garde, Suzanne C. Cannegieter, G. P. Paul Voorn, Willem Jan W. Bos, Jim E. van Steenbergen, On behalf of the Alkmaar study group, Wim G. Boersma, Menno M. van der Eerden, Dominic Snijders, On behalf of the Ovidius study group, Douwe H. Biesma, Willem Jan W. Bos, Henrik Endeman, Ewoudt M. W. van de Garde, Jan C. Grutters, Hans Hardeman, Rik Heijligenberg, Sabine C. A. Meijvis, Hilde H. Remmelts, Ger T. Rijkers, Heleen van Velzen-Blad, G. P. Paul Voorn

**Affiliations:** Department of Internal Medicine, St Antonius Hospital, Nieuwegein, The Netherlands; Department of Pulmonary Medicine, Medical Centre Alkmaar, Alkmaar, The Netherlands; Department of Clinical Pharmacy, St. Antonius Hospital, Nieuwegein, The Netherlands; Division of Pharmacoepidemiology and Clinical Pharmacology, Faculty of Science, Utrecht University, Utrecht, The Netherlands; Department of Clinical Epidemiology, Leiden University Medical Centre, Leiden, The Netherlands; Department of Medical Microbiology and Immunology, St. Antonius Hospital, Nieuwegein, The Netherlands; Centre for Infectious Disease Control, National Institute for Public Health and the Environment, Bilthoven, The Netherlands; Centre of Infectious Diseases, Leiden University Medical Centre, Leiden, The Netherlands

**Keywords:** Aetiology, Atypical pathogens, Community-acquired pneumonia, Demography, Pneumonia

## Abstract

**Background:**

Microorganisms causing community-acquired pneumonia (CAP) can be categorised into viral, typical and atypical (*Legionella* species, *Coxiella burnetii*, *Mycoplasma pneumoniae*, and *Chlamydia* species). Extensive microbiological testing to identify the causative microorganism is not standardly recommended, and empiric treatment does not always cover atypical pathogens. In order to optimize epidemiologic knowledge of CAP and to improve empiric antibiotic choice, we investigated whether atypical microorganisms are associated with a particular season or with the patient characteristics age, gender, or chronic obstructive pulmonary disease (COPD).

**Methods:**

A data-analysis was performed on databases from four prospective studies, which all included adult patients hospitalised with CAP in the Netherlands (*N* = 980). All studies performed extensive microbiological testing.

**Results:**

A main causative agent was identified in 565/980 (57.7 %) patients. Of these, 117 (20.7 %) were atypical microorganisms. This percentage was 40.4 % (57/141) during the non-respiratory season (week 20 to week 39, early May to early October), and 67.2 % (41/61) for patients under the age of 60 during this season. Factors that were associated with atypical causative agents were: CAP acquired in the non-respiratory season (odds ratio (OR) 4.3, 95 % CI 2.68–6.84), age <60 year (OR 2.9, 95 % CI 1.83–4.66), male gender (OR 1.7, 95 % CI 1.06–2.71) and absence of COPD (OR 0.2, 95 % CI 0.12–0.52).

**Conclusions:**

Atypical causative agents in CAP are associated with respectively non-respiratory season, age <60 years, male gender and absence of COPD. Therefore, to maximise its yield, extensive microbiological testing should be considered in patients <60 years old who are admitted with CAP from early May to early October.

**Trial registration:**

NCT00471640, NCT00170196 (numbers of original studies).

**Electronic supplementary material:**

The online version of this article (doi:10.1186/s12879-016-1641-9) contains supplementary material, which is available to authorized users.

## Background

Microorganisms causing community-acquired pneumonia (CAP) can be categorised into viral, typical and atypical microorganisms. Atypical microorganisms are *Legionella* species, *Coxiella* (*C.*) *burnetii* (Q-fever), *Mycoplasma* (*M.*) *pneumoniae*, and *Chlamydia* species. In clinical practice, the causative microorganism often remains unidentified since microbiological testing is not standardly performed. However, in patients with low to moderately severe CAP, treatment of first choice allegedly does not cover atypical causative microorganisms [1–3]. To start a pathogen-directed treatment, it is important to define specific conditions associated with an increased risk for atypical pathogens.

Cases of CAP are seen throughout the year, but overall incidence rises during winter months [4]. This is due to certain aetiological agents that show seasonal variation: *Streptococcus* (*S.*) *pneumoniae, Haemophilus influenzae* and respiratory viruses occur mainly during winter season [4, 5]. Of atypical microorganisms, only *Legionella* (*L.*) species and *C. burnetii* show seasonal variation, increasing during summer and during early spring in the lambing season, respectively [6–8]. Numbers of cases of *M. pneumoniae* increase during wintertime, but the incidence is relatively high during summer as well.

Concerning age, incidence of CAP is highest in young children and adults above 65 years old [9, 10]. *S. pneumoniae* is the leading causative agent in all age groups. Some atypical pathogens show an atypical age distribution. Cases of *L. pneumophila* are most commonly seen in patients aged 35 to 50 years old, psittacosis has an increased incidence in patients aged 35 to 55, and *C. burnetii* occurs most in men between 30 and 69 years old [11–13].

Furthermore, patients with chronic obstructive pulmonary disease (COPD) or positive smoking status differ in aetiology of CAP [14, 15]. Consequently, there could be a positive or negative association with these conditions and the prevalence of atypical pathogens.

Microbiological testing can be used to identify the causative microorganism and to distinguish between typical and atypical microorganisms [16]. However, guidelines do not recommend microbiological testing for patients with low to moderately severe CAP, and therefore antibiotic treatment of CAP is usually empirical [1–3]. There is no worldwide consensus on antibiotic management for CAP. The Dutch Working Party on Antibiotic Policy (SWAB) and National Institute for Health and Care Excellence (NICE) guideline recommend amoxicillin as first-choice treatment for hospitalised patients with CAP of low- to moderate severity (pneumonia severity index (PSI) classes 1–4 or CURB-65 score 0–2) and combination with a macrolide or quinolone in case of severe CAP (PSI class 5 or CURB-65 score > 2) [2, 3]. The British Thoracic Society (BTS) guideline recommends amoxicillin with macrolide combination therapy in case of moderate to severe CAP [1]. Global differences in preferred antibiotic management can partially be explained by variations in pneumococcal resistance rate between geographical regions and countries [17]. Since *S. pneumoniae* is the leading cause of CAP, the initial therapy should at least cover this microorganism. Nevertheless, beta-lactam antibiotics do not cover atypical microorganisms, leaving these pathogens theoretically uncovered by first-choice treatment for patients with low- to moderately severe CAP.

Since causative microorganisms are not extensively looked for, nor covered by antibiotic treatment in patients with CAP, it would be useful to identify specific circumstances associated with an increased risk for these pathogens as causative agent in CAP. Presence of such characteristics in a patient can then be used to determine optimal treatment. It has been shown that clinical examination, simple laboratory tests and radiographic characteristics cannot distinguish between typical and atypical microorganisms [18–20]. However, to our knowledge there is no scientific literature about seasons as risk factor for atypical pneumonias as a group. In this study, we investigated whether atypical causative microorganisms in patients with CAP are more prominent during a particular season or associated with specific patient characteristics.

## Methods

### Study design

A data-analysis was performed on databases from four prospective studies [21–24]. All studies included patients aged 18 years or older who were hospitalised with CAP in the Netherlands and gave written informed consent. Two studies were performed in the St. Antonius Hospital in Nieuwegein, from October 2004 to August 2006 [23] and from November 2007 to September 2010 [21]. The other studies were performed in Medical Center Alkmaar from December 1998 to November 2000 [24] and August 2005 to July 2008 [22]. On all patients, extensive microbiological investigations for pathogen identification was performed such as blood cultures, sputum cultures, urine antigen tests for *L. pneumophila* serogroup 1 and *S. pneumoniae*, and paired complement fixation test [21] or ELISA test [22–24] for *M. pneumoniae, C. burnetii, Chlamydia* species and the viruses adenovirus, influenza virus A and B, parainfluenza virus 1, 2 and 3, and the respiratory syncytial virus. A four‐fold increase in antibody titre was considered as positive. Two studies performed polymerase chain reaction (PCR) for atypical pathogens and respiratory viruses [21, 23]. Further details are described elsewhere [21–24].

The following data were used from these datasets: date of admission, age, gender, antibiotics treatment before hospitalisation, duration of antibiotic therapy, presence of COPD, smoking status, whether the patient was living in a nursing home, PSI at admission, mortality within 30 days and main causative pathogen.

### Definition of season and outcome

Date of admission was classified to be either in the respiratory season (week 40 to 19; from early October to early May) or the non-respiratory season (week 20 to 39). This classification was chosen because we expected atypical microorganisms to be associated with non-respiratory season, considering their known seasonal variation, and because the format is easy to use in daily practice. Main causative agents were grouped in three categories: 'atypical', 'other' and 'unknown'. Data-analysis was performed using ‘atypical vs. other pathogen’ as outcome. The category ‘atypical’ consisted of *Legionella* species, *M. pneumoniae*, *Chlamydia* species and *C. burnetii* as causative agents. The category ‘other’ consisted of all other bacterial and viral causative microorganisms. The category ‘unknown’ consisted of patients with CAP in whom the causative agent remained unidentified after microbiological investigation. These unidentified cases were excluded from the primary analysis since it is unknown whether the proportion of atypical causative pathogens is lower, higher or equal to the identified group.

### Statistical analysis

Data management and statistical analysis were performed using IBM SPSS Statistics for Windows, Version 22.0. Categorical variables were summarized as percentages, non-categorical variables were summarized as mean (standard deviation) or median (interquartile range) as appropriate. Differences between categories were observed by means of Pearson’s chi-squared test for parametrical variables, versus Kruskall-Wallis test for non-parametrical samples.

To determine an age cut-off for further analyses, the percentage of atypical pathogens in patients younger and older than all ages between 40 and 70 years old was calculated. A cut-off point at age 60 was chosen based on these results.

Univariable logistic regression analysis was performed for the following parameters: respiratory season, age <60, male gender, presence of COPD and smoking. Variables significant in univariable analysis and variables expected to be associated with atypical causative agents were selected for multivariable analysis. Potential interactions between age and presence of COPD were investigated using interaction terms in logistic regression analysis. Smoking was excluded from multivariable analysis due to 243 missing values.

Several sensitivity analyses were performed. In the first sensitivity analyses viral pathogens were excluded. The second analysis indicated all unknown pathogens as not atypical; the third analysis indicated all unknown pathogens as atypical. A fourth analysis excluded *Legionella* CAPs in 1999 and *C. burnetii* CAPs in 2009, based on the outbreaks in those years of inclusion [6, 8]. Another sensitivity analysis excluded all *Legionella* CAPs. Also, an analysis was performed including exclusively the two studies that performed PCR to detect atypical pathogens and respiratory viruses [21, 23]. Last, extra analyses were performed excluding patients who were treated with antibiotics before hospitalisation and patients with a severe CAP (PSI score V), respectively.

## Results

### Aetiology and baseline characteristics

**Fig. 1:**
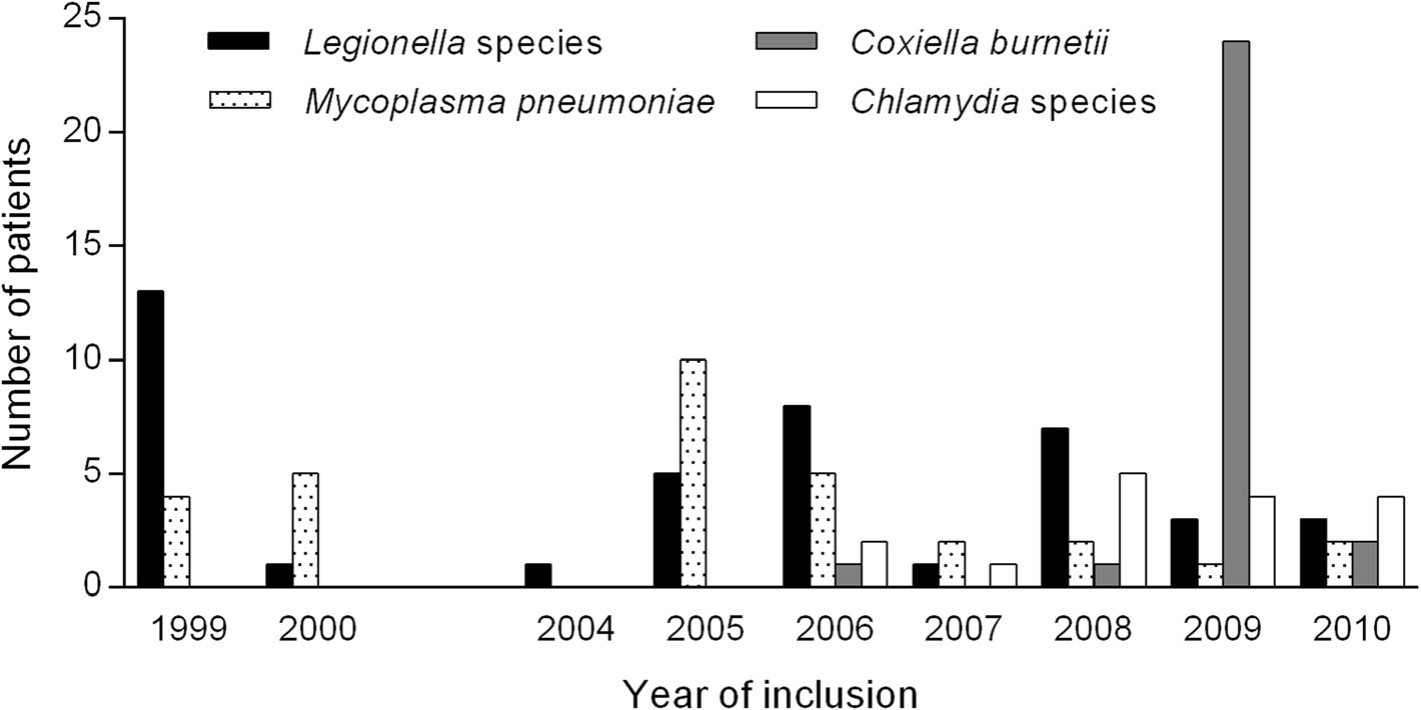
Number of atypical pathogens per year of inclusion

In total 980 patients were included in the analysis, including all patients from the original four databases. Median age was 67 years (IQR 52–78) and 57.2 % was male. These distributions did not significantly differ between the four studies. Table 1 shows all baseline characteristics of the included studies. In 565/980 (57.7 %) patients the causative microorganism of CAP was identified. Atypical microorganisms were causative pathogens in 117/980 patients (11.9 %); 35.9 % were cases with *Legionella* species (42/117), 26.5 % with *M. pneumoniae* (31/117), 23.9 % with *C. burnetii* (28/117), and 13.7 % with *Chlamydia* species (16/117). Figure 1 shows the number of every atypical pathogen per year of inclusion.

**Table 1:** Baseline characteristics and aetiology per study of all 980 patients

	All patients (*n* = 980)	Meijvis [21] (*n* = 304)	Endeman [23] (*n* = 201)	Snijders [22] (*n* = 213)	Vd Eerden [24] (*n* = 262)
Demographic characteristics	No.	No.	No.	No.	No.
Male gender (%)	561 (57.2)	171 (56.3)	124 (61.7)	124 (58.2)	142 (54.2)
Age in years, median (IQR)	67 (52–78)	66.5 (51–79)	68 (51.5–76)	65 (51.5–80)	68 (52–78)
COPD (%)	236 (24.2)	34 (11.2)	64 (31.8)	43 (20.3)	95 (36.7)
Smoking (%)	211 (28.6)	81 (29.2)	^a^	56 (26.8)	74 (29.5)
Nursing home resident (%)	29 (3.0)	16 (5.3)	3 (1.5)	4 (1.9)	6 (2.3)
PSI class IV or V (%)	435 (44.4)	142 (46.7)	84 (41.8)	94 (44.1)	115 (43.9)
30-Day mortality (%)	67 (6.8)	18 (5.9)	8 (4.0)	12 (5.6)	29 (11.1)
Aetiology (%)					
* Streptococcus pneumoniae*	282 (28.8)	64 (21.1)	60 (29.9)	78 (36.6)	80 (30.5)
* Haemophilus influenzae*	55 (5.6)	9 (3.0)	18 (9.0)	7 (3.3)	21 (8.0)
* Legionella* species	42 (4.3)	12 (3.9)	8 (4.0)	8 (3.8)	14 (5.3)
* Mycoplasma pneumoniae*	31 (3.2)	5 (1.6)	4 (2.0)	13 (6.1)	9 (3.4)
* Coxiella burnetii*	28 (2.9)	27 (8.9)	1 (0.5)	0 (0)	0 (0)
Influenza A/B virus	25 (2.6)	7 (2.3)	6 (3.0)	3 (1.4)	9 (3.4)
* Staphylococcus aureus*	21 (2.1)	3 (1.0)	6 (3.0)	2 (0.9)	10 (3.8)
* Chlamydia* species	16 (1.6)	14 (4.6)	2 (1.0)	0 (0)	0 (0)
Adenovirus	6 (0.6)	1 (0.3)	1 (0.5)	2 (0.9)	2 (0.8)
Other bacteria	38 (3.9)	15 (4.9)	12 (6.0)	5 (2.3)	6 (2.3)
Other viruses	21 (2.1)	11 (3.6)	9 (4.5)	0 (0)	1 (0.4)
Unidentified	415 (42.3)	136 (44.7)	74 (36.8)	95 (44.6)	110 (42.0)

Results are given as number and percentage, unless mentioned otherwise

*Abbreviations*: *COPD* chronic obstructive pulmonary disease, *ICU* intensive care unit, *IQR* interquartile range, *PSI* pneumonia severity index

^a^Indicates all missing values

Further analyses were performed on the three aetiologic categories (atypical, other pathogen and unknown). Baseline characteristics of the patients in these three categories are shown in Table 2. The variables age, COPD, smoking, nursing home resident and severity of pneumonia (according to PSI class) differed significantly between the three categories.

**Table 2:** Demographic characteristics and outcome of 980 patients hospitalised with community-acquired pneumonia

	Total (*n* = 980)	Atypical (*n* = 117)	Other pathogen (*n* = 448)	Unknown (*n* = 415)
Male gender (%)	561 (57.2)	77 (65.8)	250 (55.8)	234 (56.4)
Age in years (IQR)	67 (52–78)	52 (41.5–67)	68 (54–78)	69 (55–80)*
COPD (%)^a^	236 (24.1)	9 (7.7)	135 (30.3)	92 (22.3)*
Smoking (%)^b^	211 (28.6)	38 (40.4)	93 (29.3)	80 (24.5)*
Nursing home resident (%)^c^	29 (3.0)	0 (0.0)	8 (1.8)	21 (5.1)*
PSI class IV or V (%)	435 (44.4)	38 (32.5)	219 (48.9)	178 (42.9)*
30-Day mortality (%)	67 (6.8)	6 (5.1)	33 (7.4)	28 (6.7)

Results are given as number (percentage) or median (interquartile range)

*Abbreviations*: *COPD* chronic obstructive pulmonary disease, *ICU* intensive care unit, *IQR* interquartile range, *PSI* pneumonia severity index

*Indicates a significant difference between the three groups (*p*-value <0.05). ^a^Indicates four missing values, ^b^Indicates 243 missing values, ^c^Indicates 10 missing values

### Seasonal and age variations

**Fig. 2:**
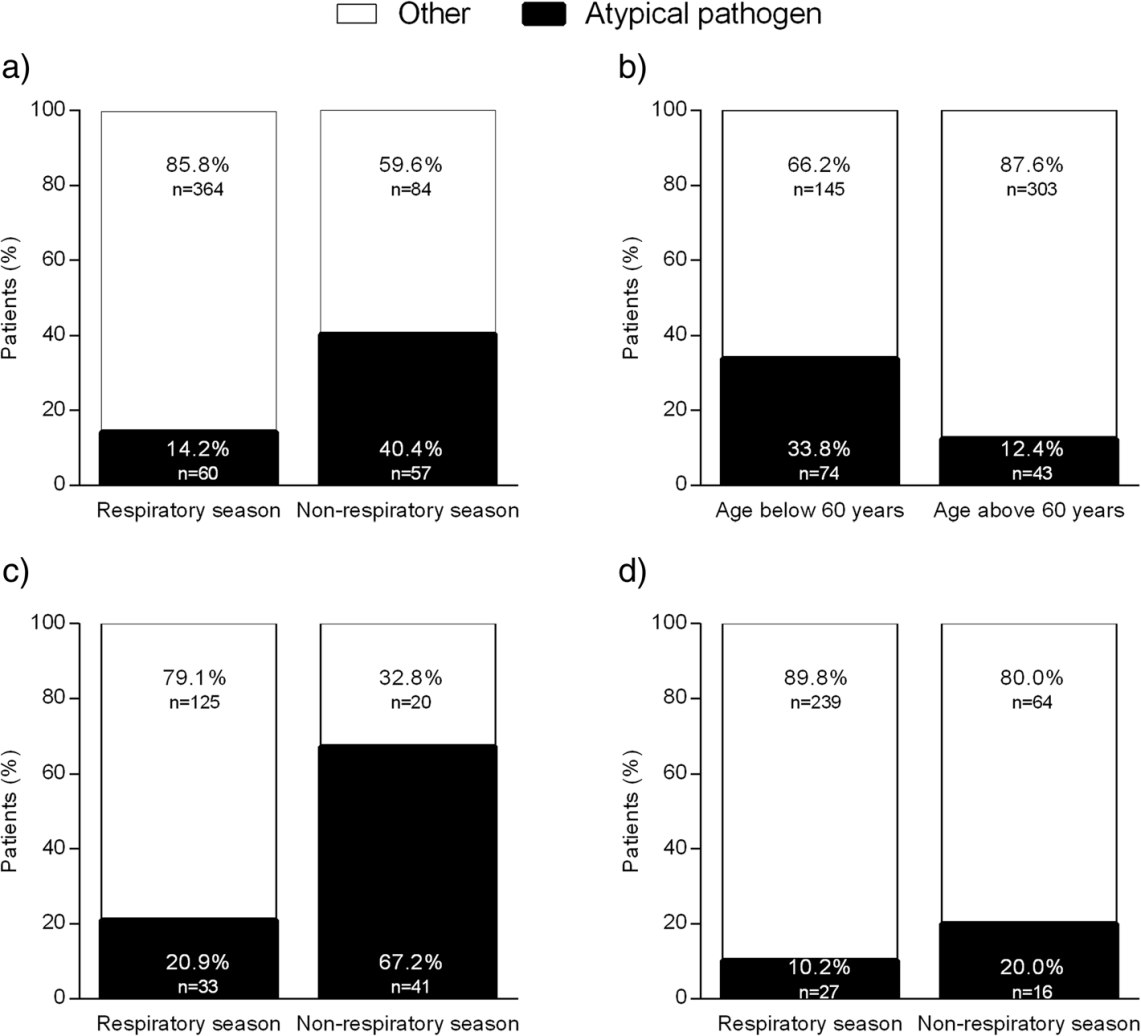
Percentage of atypical pathogen. **a** Distribution of pathogens during the different seasons. **b** Distribution of pathogens related to age. **c** Distribution of pathogens within season for patients < 60 years. **d** Distribution of pathogens within season for patients ≥ 60 years

Of all identified microorganisms, the percentage of atypical pathogens was 20.7 % (117/565). Forty percent (40.4 %, *n* = 57) of CAP cases during the non-respiratory season were atypical, compared to 14.2 % (*n* = 60) during the respiratory season (Fig. 2a).

For CAP in patients under 60 years of age, 33.8 % (*n* = 74) was caused by an atypical pathogen, compared to 12.4 % (*n* = 43) for patients ≥ 60 years (Fig. 2b).

Combining seasonal effect and age, incidence of CAP caused by atypical microorganisms during the non-respiratory season was 67.2 % (*n* = 41) in patients younger than 60 years. This percentage was 20 % in patients ≥ 60 years (*n* = 16, Fig. 2c and d).

### Logistic regression analysis

In univariable and multivariable logistic regression analysis, non-respiratory season, age <60, and absence of COPD were significantly associated with an atypical causative agent. No significant interaction was found between age and COPD (p:0.61). Male gender was nearly significant in univariable analysis (OR 1.5, 95 % 1.00–2.33, p:0.052), and significant in multivariable analysis (OR 1.7, 95 % 1.06–2.71). Too little patients were living in a nursing home to include this variable in the analyses. Table 3 shows all ORs and 95 % CIs.

**Table 3:** Univariable and multivariable logistic regression analysis of atypical versus other pathogens with cases with unknown aetiology excluded

	Univariable analysis		Multivariable analysis	
Parameter	OR	95 % CI	OR	95 % CI
Non-respiratory season	4.1	2.67–6.35**	4.3	2.68–6.84**
Age <60 years	3.6	2.35–5.50**	2.9	1.83–4.66**
Male gender	1.5	1.00–2.33	1.7	1.06–2.71*
COPD^a^	0.2	0.09–0.39**	0.2	0.12–0.52**
Smoking^b^	1.6	1.01–2.64*		Excluded

*Abbreviations*: *OR* odds ratio, *CI* confidence interval, *COPD* chronic obstructive pulmonary disease

*Indicates a *p*-value <0.05; **Indicates a *p*-value < 0.001

^a^Indicates four missing values; ^b^Indicates 243 missing values

### Sensitivity analyses

When viral pathogens were excluded from multivariate analysis, odds ratios did not essentially change (see Table 4). Similar results were found when all unknown pathogens were indicated as not atypical. When all unknown pathogens were indicated as atypical, the OR for non-respiratory season remained significantly elevated, ORs for age <60 and male gender remained elevated but became non-significant. All ORs can be found in Table 4.

**Table 4:** Three multivariable logistic regression sensitivity analyses: 1) Excluding viral pathogens; 2) Cases with unknown aetiology being indicated as not atypical; and 3) Cases with unknown aetiology being indicated as atypical

	1) Atypical vs. other (viral cases excluded)		2) Atypical vs. other and unknown		3) Atypical and unknown vs. other	
Parameter	OR	95 % CI	OR	95 % CI	OR	95 % CI
Non-respiratory season	4.2	2.58–6.72**	3.0	1.95–4.47**	2.0	1.47–2.76**
Age <60 years	2.7	1.69–4.36**	3.0	1.97–4.59**	1.0	0.75–1.35
Male gender	1.7	1.09–2.81*	1.6	1.02–2.41*	1.2	0.91–1.56
COPD^a^	0.2	0.11–0.48**	0.3	0.15–0.65*	0.5	0.37–0.70**

*Abbreviations*: *OR* odds ratio, *CI* confidence interval, *COPD* chronic obstructive pulmonary disease

*Indicates a *p*-value <0.05; **Indicates a *p*-value < 0.001

^a^Indicates four missing values

The sensitivity analysis excluding all *Legionella* cases in 1999 and *C. burnetii* cases in 2009 showed similar results (see Table 5). The analyses excluding all *Legionella* CAPs and the two studies that did not use PCR methods all showed non-respiratory season and age < 60 to be even stronger predictors for atypical CAP (see Table 5).

**Table 5:** Two multivariable logistic regression sensitivity analyses with: 1) Outbreaks excluded; 2) All *Legionella* cases excluded; 3) Only two studies that used PCR methods

	1) Outbreaks excluded		2) *Legionella* cases excluded		3) PCR based studies	
	OR	95 % CI	OR	95 % CI	OR	95 % CI
Non-respiratory season	4.2	2.38–7.30**	5.4	3.02–9.64**	8.3	4.35–15.80**
Age <60 years	4.2	2.38–7.30**	6.1	3.24–11.55**	3.7	1.93–7.06**
Male gender	1.8	1.02–3.05*	1.7	0.94–3.00	1.4	0.74–2.68
COPD^a^	0.2	0.08–0.54*	0.1	0.04–0.51*	0.2	0.07–0.71*

*Abbreviations*: *OR* odds ratio, *CI* confidence interval, *COPD* chronic obstructive pulmonary disease

*Indicates a *p*-value <0.05; **Indicates a *p*-value < 0.001

^a^Indicates four missing values

Excluding PSI class 5 resulted in remaining significance for non-respiratory season, age <60 and COPD. The OR for male gender was higher than 1 (1.5), but not statistically significant. Non-respiratory season, age <60 and COPD all remained significant predictors for an atypical causative agent in the analysis excluding patients who were treated with antibiotics before hospitalisation. Results from the analysis excluding respectively patients with PSI class 5 and patients who used antibiotics before hospitalisation can be found in Additional file 1: Table S1.

## Discussion

In this analysis of 980 patients hospitalised with CAP in whom extensive microbiological testing was performed, we found non-respiratory season, age < 60, male gender and absence of COPD to be associated with an atypical microorganism as causative agent. The proportion of CAP cases caused by atypical microorganisms was much higher during the non-respiratory season than in respiratory season (40.4 % vs. 14.2 %). This proportion was even higher in patients below 60 years in non-respiratory season (67.2 %).

To our knowledge, there is no scientific literature about season as a risk factor for atypical pneumonias. Factors that indicate an association with pneumonia caused by this group of pathogens can optimize epidemiologic knowledge. Since none of the atypical pathogens are theoretically covered by empiric antibiotic treatment in non-severe pneumonia, this knowledge can possibly reduce treatment failure in this pathogen group. In literature, there is controversy regarding the need to use antibiotics covering atypical pathogens in the initial treatment of patients with CAP. Recent studies show no differences in clinical efficiency, nor differences in mortality between antibiotic treatment with and without atypical coverage, meaning that these atypical infections might resolve spontaneously under inadequate antibiotic therapy [25–27]. Mostly, this relates to the comparison of quinolone monotherapy with beta-lactam monotherapy. However, these studies describe empirical treatment of CAP, regularly without extensive microbiological testing and therefore the causing pathogen is often not identified [25, 26]. It is unknown if outcome of CAPs caused by an atypical pathogen is improved if adequate antibiotic coverage is started immediately on admission. In the CAP-START study, beta-lactam therapy was non-inferior to beta-lactam plus macrolide, but 8.1 % of the patients who were empirically treated with beta-lactams did deviate from the study protocol due to the need for atypical coverage. Overall, 38.7 % of the patients in the beta-lactam group received atypical antibiotic coverage during admission [27].

For observing seasonal variations, we used the format of calendar respiratory season vs. non-respiratory season (from early May to early October). This format was used because we expected the proportion of atypical microorganisms to be elevated in the non-respiratory season. Furthermore, the format non-respiratory season is easy to use in daily practice. The use of other formats such as astronomical seasons or influenza-epidemic could provide additional information on the epidemiology of CAP in the Netherlands. We performed additional analyses using these formats, showing the highest percentage of atypical CAPs during summer and lowest incidence during winter (data not shown). Using respiratory vs. non-respiratory season showed highest predictive value.

The main limitation of this study is the fact that the prevalence of atypical microorganisms could have been over- or underestimated due to several reasons. First, it is impossible to assess the proportion of atypical causative agents in the unidentified group. It is suggested that the majority of unidentified CAP cases is caused by *S. pneumoniae*, but it is unknown whether the proportion of atypical causative pathogens is lower, higher or equal to the identified group [28]. As only the distribution in the group of identified cases is evident, data-analysis was performed using ‘atypical vs. other proven causative agent’ as outcome, hereby excluding the unidentified cases. This could have led to either over- or underestimation. However, the sensitivity analyses defining unidentified cases as not atypical showed small differences. Since it is debatable to appoint viral pathogens as main CAP causing microorganism, we performed an analysis excluding viral cases. This analysis showed similar results for all used variables.

A second limitation is that all CAP severities were included in the analysis. Treatment regimens differ worldwide, but it is usually recommended to cover atypical causative microorganisms in patients with severe CAP. Because we aimed to identify patient characteristics associated with atypical causative microorganisms in all cases of CAP, severe CAPs were not excluded from analyses. To validate our results for patients with low-to moderately severe CAP, the extra analysis was performed excluding patients with PSI class 5, which showed similar results.

Third, we used data from four different studies, in which not exactly the same microbiological tests were performed. Two studies did not use PCR, whereas this test is known to be more sensitive for diagnosing atypical bacteria compared to conventional testing [29]. In the studies that performed PCR, 17.4 % of atypical microorganisms was identified with PCR alone. It is therefore possible that atypical microorganisms were underrepresented in the studies without PCR [22, 24]. Including only the two studies that used PCR methods showed elevated ORs for non-respiratory season and age <60. All four studies did perform two-fold serological testing for antibodies against atypical microorganisms, but these tests have shortcomings in sensitivity and specificity in diagnosing atypical microorganisms [30]. Pneumococcal antigen testing has not been consequently performed in all studies. Antigen detection performed in non-urine specimens may increase the diagnostic yield of pneumococcal infections, but was performed in only two studies [22, 24, 31].

Finally, some patients had already been treated with antibiotics before admission. Performing PCR and serology for atypical pathogens while using only cultures for other aetiology could have led to an overestimation of atypical pathogens, since cultures are influenced by antibiotic pre-treatment whereas serology and PCR are not. We performed an analysis excluding all patients who were treated with antibiotics before hospital admission, which did not change the results.

A strength of this study is the large number of identified atypical pathogens, enabling us to identify significant predictors for CAP caused by atypical pathogens. Furthermore, extensive microbiological testing was performed for every patient, hereby providing a detailed impression on epidemiology of CAP. Our results are applicable for hospitalised patients, but might be extrapolated to general practice. Several studies have shown that microbiological aetiology of CAP differs between hospital and ambulatory setting, with a higher incidence of atypical pathogens and respiratory viruses in mild cases of CAP in ambulatory setting [10, 32, 33]. This suggests that for general practitioners respiratory season, gender and age might be adequate indicators to identify pneumonia patients in whom microbiologic testing needs to be considered or in whom antibiotic treatment (if required) should not be selected purely on the existing first choice regimen. Further research is needed to explore whether there are differences in clinical outcome for patients under the age of 60 presenting during non-respiratory season when treated with antibiotics with and without immediate atypical coverage. With regard to the results presented in this study, we would recommend to perform extensive microbiological testing in male patients, younger than 60 years old, who are admitted with CAP during non-respiratory season in order to optimise epidemiologic knowledge.

## Conclusions

Atypical causative agents in CAP are associated with respectively non-respiratory season, age <60 years, male gender and absence of COPD. Therefore, extensive microbiological testing should be considered in patients <60 years old who are admitted with CAP from early May to early October.

## Abbreviations

95 % CI, 95 % confidence interval; BTS, British Thoracic Society; *C. Burnetii*, *Coxiella burnetii*; CAP, community-acquired pneumonia; COPD, chronic obstructive pulmonary disease; ELISA, enzyme-linked immuno sorbent assay; *L. pneumophila*, *Legionella pneumophila*; *M. pneumoniae*, *Mycoplasma pneumoniae*; NICE, National Institute for Health and Care Excellence; OR, odds ratio; PCR, polymerase chain reaction; PSI, pneumonia severity index; *S. pneumoniae*, *Streptococcus pneumoniae*; SWAB, Dutch Working Party on Antibiotic Policy

## Additional file

Additional file 1: Table S1. Extra multivariable logistic regression analyses, excluding: 1) Pneumonia severity index class V; and 2) Patients who were treated with antibiotics before hospitalisation. (DOC 37 kb)
