# Supplementary material for: Atypical aetiology in patients hospitalised with community-acquired pneumonia is associated with age, gender and season; a data-analysis on four Dutch cohorts
Source: BMC Infect Dis. 2016 Jun 17;16:299. doi: 10.1186/s12879-016-1641-9 (PMC4912822; doi:10.1186/s12879-016-1641-9)
Supplement: Additional file 1: Table S1. — Extra multivariable logistic regression analyses, excluding: 1) Pneumonia severity index class V; and 2) Patients who were treated with antibiotics before hospitalisation. (DOC 37 kb) [file 12879_2016_1641_MOESM1_ESM.doc]

**Additional file belonging to the manuscript entitled**

**‘**Atypical aetiology in patients hospitalised with community-acquired pneumonia is associated with age, gender and season; a data-analysis on four Dutch cohorts**.’**

**Authors**: Vivian M Raeven; Simone MC Spoorenberg; Wim G. Boersma; Ewoudt MW van de Garde; Suzanne C Cannegieter; GP (Paul) Voorn; Willem Jan W Bos; Jim E van Steenbergen; and the Ovidius and Alkmaar study groups.

**TABLE S1. Extra multivariate logistic regression analyses, excluding: 1) Pneumonia severity index class V; and 2) Patients who were treated with antibiotics before hospitalisation.**

|  | 1) Excluding  PSI class V | | 2) Excluding antibiotics pre-treatment | |
| --- | --- | --- | --- | --- |
|  | OR | 95% CI | OR | 95% CI |
| Non-respiratory season | 4.3 | 2.66-7.07** | 4.4 | 2.52-7.65** |
| Age <60 years | 2.6 | 1.57-4.20** | 1.9 | 1.10-3.40* |
| Male gender | 1.5 | 0.95-2.48 | 1.4 | 0.82-2.51 |
| COPD^ | 0.3 | 0.12-0.56** | 0.2 | 0.09-0.56** |

Abbreviations: OR, odds ratio; CI, Confidence Interval; COPD, chronic obstructive pulmonary disease. * Indicates a *p*-value <0.05; ** Indicates a *p*-value<0.001; ^ Indicates four missing values.
